# Co-Doped Magnesium Oxychloride Composites with Unique Flexural Strength for Construction Use

**DOI:** 10.3390/ma15020604

**Published:** 2022-01-14

**Authors:** Anna-Marie Lauermannová, Ondřej Jankovský, Michal Lojka, Ivana Faltysová, Julie Slámová, Milena Pavlíková, Adam Pivák, Šimon Marušiak, Zbyšek Pavlík, Martina Záleská

**Affiliations:** 1 Department of Inorganic Chemistry, Faculty of Chemical Technology, University of Chemistry and Technology, Technická 5, 166 28 Prague, Czech Republic; Anna-Marie.Lauermannova@vscht.cz (A.-M.L.); ondrej.jankovsky@vscht.cz (O.J.); michal.lojka@vscht.cz (M.L.); ivana.faltysova@vscht.cz (I.F.); Julie.Slamova@vscht.cz (J.S.); 2 Department of Materials Engineering and Chemistry, Faculty of Civil Engineering, Czech Technical University in Prague, Thákurova 7, 166 29 Prague, Czech Republic; milena.pavlikova@fsv.cvut.cz (M.P.); adam.pivak@fsv.cvut.cz (A.P.); simon.marusiak@fsv.cvut.cz (Š.M.); pavlikz@fsv.cvut.cz (Z.P.)

**Keywords:** co-doped magnesium oxychloride cement, graphene oxide, oxidized MWCNTs, mechanical and physical parameters, microstructure, morphology and phase composition

## Abstract

In this study, the combined effect of graphene oxide (GO) and oxidized multi-walled carbon nanotubes (OMWCNTs) on material properties of the magnesium oxychloride (MOC) phase 5 was analyzed. The selected carbon-based nanoadditives were used in small content in order to obtain higher values of mechanical parameters and higher water resistance while maintaining acceptable price of the final composites. Two sets of samples containing either 0.1 wt. % or 0.2 wt. % of both nanoadditives were prepared, in addition to a set of reference samples without additives. Samples were characterized by X-ray diffraction, scanning electron microscopy, Fourier-transform infrared spectroscopy, and energy dispersive spectroscopy, which were used to obtain the basic information on the phase and chemical composition, as well as the microstructure and morphology. Basic macro- and micro-structural parameters were studied in order to determine the effect of the nanoadditives on the open porosity, bulk and specific density. In addition, the mechanical, hygric and thermal parameters of the prepared nano-doped composites were acquired and compared to the reference sample. An enhancement of all the mentioned types of parameters was observed. This can be assigned to the drop in porosity when GO and OMWCNTs were used. This research shows a pathway of increasing the water resistance of MOC-based composites, which is an important step in the development of the new generation of construction materials.

## 1. Introduction

The recent advances in the research of the magnesium oxychloride cement (MOC) [1,2] show a promising direction in the field of alternative construction materials. As non-hydraulic binder prepared from light-burnt magnesia, MOC is usually considered as an environmentally sustainable and energy efficient construction material [3]. The material itself provides high values of compressive and flexural strength [4,5]; it is fireproof [6] and it has very short setting time [7]. Its ability to bond a wide range of additives makes it possible to enhance its properties by using various fillers and to create materials with very specific mechanical, physical, chemical and functional properties, which are applicable in many branches of the construction industry. It is possible to use great volumes of secondary fillers, such as diatomite [8], fly ash [9,10], glass powder [11], wood aggregates [12,13], lunar regolith simulant [14] or others [15], in order to create an eco-friendly material, while maintaining the high mechanical strength and stiffness.

The production of MOC is quite simple and depending on the molar ratio of the raw materials and the conditions of the synthetic procedure, such as temperature, a variety of compounds in the system MgO-MgCl_2_-H_2_O can be synthesized [16,17]. The most common and used phase of this system is MOC phase 5 (also known as MOC 5-1-8), whose formula is 5Mg(OH)_2_∙MgCl_2_∙H_2_O. This phase crystallizes in the form of needle-shaped whiskers [18]. The interlocking of these crystals is one of the possible causes of the high strength of the binder [19]. The properties of MOC are also influenced by its ability to absorb CO_2_ from the atmosphere [20,21] which results in the formation of magnesium carbonate phases on the surface of MOC.

The main issue of MOC is its low water resistance [22,23]. The decomposition of MOC in the water environment and the reaction of the present phases with water which forms brucite have been previously described in the literature [24,25]. During this process, the mechanical strength decreases rapidly and leads to leaching of magnesium chloride, which creates a corrosive environment. Therefore, such material is not applicable in combination with steel reinforcement [26,27]; alternative reinforcing materials must be researched [28]. The issue of the low water resistance was previously studied by many researchers and various approaches have been suggested. Li et al. [29] have described the process of using phosphoric acid and soluble phosphates in the amount of 0.5–1.0 wt. % of the MOC phase, resulting in a very significant increase in the water resistance. Similarly, citric acid [30] and tartaric acid [31,32] also showed improvement on the water resistance of MOC. The use of fly ash as an additive for increasing the water resistance was described by Wu et al. [33]; however, the influence of the dosage of the fly ash on the mechanical properties of the prepared composites was negative and quite significant. Another approach was used by He et al. [34], who suggested the use of incinerated sewage sludge ash. The disadvantage of this method is in the varying composition of the ash in different locations and, therefore, different effects on the water resistance. A possible solution to this problem lies in the area of the carbon-based nanomaterials.

The general use of carbon-based nanomaterials in the construction composites is based on the addition of a small amount of the nanomaterial into the matrix while obtaining large enhancement of the material properties. This originates in the extraordinary properties of the C-based nanomaterials, namely their high mechanical strength [35], great electrical and thermal conductivity [36], and outstanding optical properties [37]. Graphene and its derivatives belong to the group of 2D carbon nanomaterials. The functionalization of graphene and formation of its derivative has been thoroughly studied in the past years. It can be modified with sulfur, fluorine, boron, nitrogen and other elements [38,39,40,41,42,43]. The multi-walled carbon nanotubes (MWCNTs) and their derivatives are representatives of the 1D group of carbon nanomaterials. Analogically to graphene, MWCNTs can be modified to create many derivatives with various functional groups [44,45,46,47,48].

The use of graphene and carbon nanotubes in ordinary construction composites is limited by their difficult dispersion in water, which is caused by their hydrophobicity [49]. The hydrophobic nature of these materials can be affected by their functionalization, and therefore the dispersion of such materials in the composite matrix is much easier. The process of functionalization of these nanoadditives is usually based on their reaction with strong acids and formation of their oxidized analogues [50,51]. The oxidized analogues contain hydroxyl (-OH), carboxyl (-COOH) and other groups, which can react with the matrix itself and contribute to the mechanical properties of the final composite [52,53].

The use of carbon-based nanoadditives in MOC as a mechanical and physical properties-enhancement agent has been previously described in the literature. It has been proven that a very small amount of graphene (G), graphene oxide (GO), multi-walled carbon nanotubes (MWCNTs) or their oxidized analogues (OMWCNTs), can improve the mechanical and physical properties of the MOC matrix [54,55].

Using GO and OMWCNTs as additives in MOC should result in the formation of various types of interactions between their atoms and the magnesium ions in the MOC matrix. This is caused by the reaction between such ions and the oxygen-containing groups of the oxidized nanoadditives [56]. In the previous studies, the interactions between magnesium ions and GO have been divided into two groups: (1) bridging the edges of the sheets through carboxylate chelates to the metal and (2) intercalating between the basal planes through either weak alkoxide or dative bonds from carbonyl and hydroxyl groups; however, it has been shown that the latter type are weak and can be removed by water rinsing. Moreover, as GO contains reactive epoxy groups, its exposure to Lewis acidic divalent metal ions such as Mg^2+^ may lead to ring-opening of such groups. During this process, new types of C-O bonds may form [57,58]. The interactions between OMWCNTs and magnesium ions are mainly chemical; however, the electrostatic interactions and sorption-precipitation interactions also occur at a lower rate. The origin of such interaction is caused by the nature of the oxygen-containing groups, which induce negative charge on the OMWCNT’s surface, resulting in donation of the single pair of electrons from the oxygen to the magnesium ions [59].

In this study, a combined influence of graphene oxide and OMWCNTs on the properties of MOC matrix was evaluated. The novelty of the paper lies in fact that the effect of co-doping of MOC matrix by a blend of 1-D and 2-D oxidized carbon-based nanoadditives has not been analyzed yet, although it can bring potential improvement in technical and functional parameters of multi-scale MOC composites for construction use. Based on the required mechanical and material properties, according amounts of both nanoadditives were chosen and used for the preparation of a novel composite based on reactive magnesia. The basic micro- and macro-structural parameters, as well as the mechanical, hygric and thermal properties, were studied in order to obtain a complex insight into the problems of the nano-doping of MOC with oxidized forms of carbon-based nanoadditives.

## 2. Materials and Methods

The studied composites were prepared using MgCl_2_·6H_2_O (>99%, Lach-Ner Ltd., Neratovice, Czech Republic), light MgO powder (>98%, Penta Ltd., Prague, Czech Republic), graphene oxide (lateral size 0.2–10 µm, thickness 2 nm, ACS Material, LLC., Pasadena, CA, USA) and functionalized multi-walled carbon nanotubes (TNIMC8 with declared purity >95 wt. %, length < 10 µm, TimesNano, Chengdu, China). Both carbon-based nanomaterials were analyzed before their addition to MOC-based composites. The BET specific surface was 47.1 m^2^·g^−1^ for graphene oxide and 76.9 m^2^·g^−1^ for OMWCNTs.

**Figure 1:**
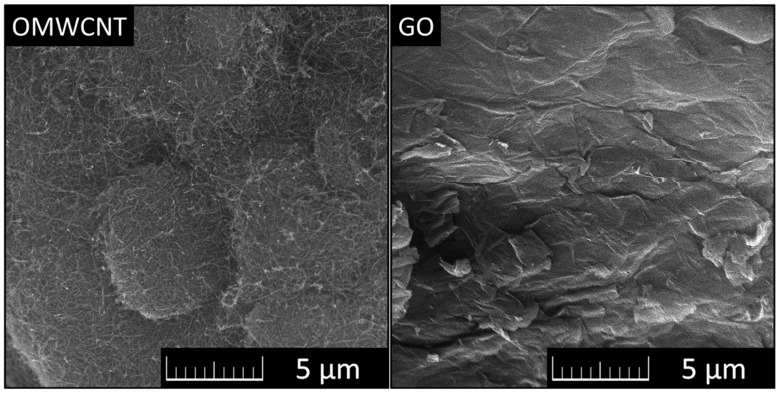
Micrographs of OMWCNTs (**left**) and GO (**right**) observed using SEM.

Micrographs of OMWCNTs and GO can be seen in Figure 1.

Elemental composition of both nanoadditives is shown in Table 1.

**Figure 2:**
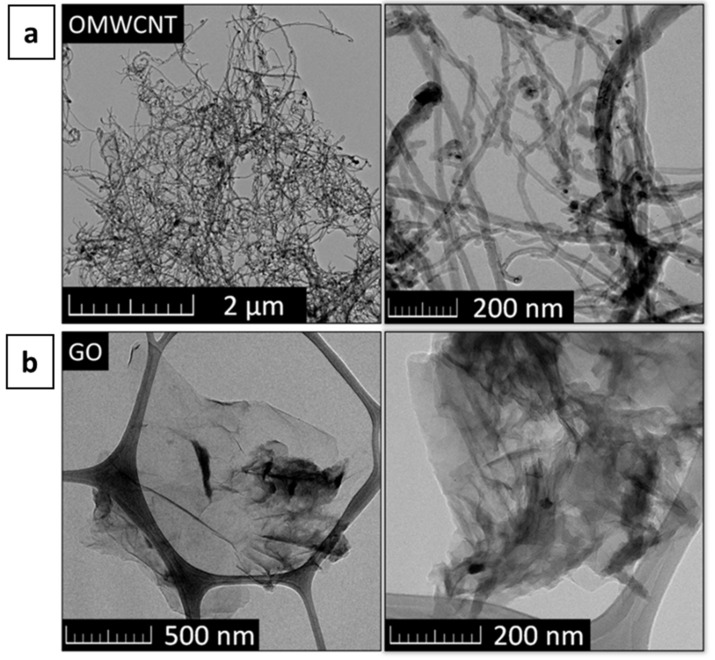
TEM image of (**a**) OMWCNTs and (**b**) GO.

The obtained microstructure of OMWCNTs and Graphene Oxide (GO) is shown in Figure 2.

The composition of the prepared samples is introduced in Table 2. In the formulation of the mixtures, MgO was added in excess, its unreacted part served as filler. MOC-REF is a reference mixture without nanoadditives. In samples marked GO-OMWCNT, the graphene oxide (GO) and oxidized multi-walled carbon nanotubes (OMWCNTs) were used in the amount of 0.1 wt. % (MOC-GO-OMWCNT-0.1) and 0.2 wt. % (MOC-GO-OMWCNT-0.2) of the whole MOC mixture.

MgCl_2_·6H_2_O was dissolved in tap water. GO and OMWCNTs were dosed and dispersed together in the part of the prepared MgCl_2_ water solution using a homogenizer UltraTurrax T-18 (IKA, Staufen im Breisgau, Germany). This first step of homogenization took 5 min and was realized at 11,000 rpm. The obtained suspension was transferred to a planetary type mixer (ELE) with the rest of MgCl_2_ solution, and the MgO powder was added. The second homogenization in ELE took 5 min. The fresh mixtures were then cast into steel molds with dimensions of 40 mm × 40 mm × 160 mm, unmolded after 24 h, and cured in the air atmosphere laboratory at temperatures of (23 ± 2) °C and relative humidity of (50 ± 5)%. After 27 days, the samples were submitted to the particular tests.

For the fresh mixtures, rheological parameters such as dynamic viscosity *η* (Pa·s) and shear stress *τ* (Pa) as functions of velocity gradient were measured in order to document possible effect off MOC co-doping on the changes of its workability. The measurement itself was performed by the use of the rotation viscometer HAAKE Viscometer E (Thermo Fisher Scientific, Waltham, MA, USA) equipped with a rotary spindle R3 type.

X-ray powder diffraction (XRD) was carried out using a Bruker D2 Phaser (Bruker AXS GmbH, Karlsruhe, Germany), a powder diffractometer with Bragg–Brentano geometry, applying CuKα radiation (λ = 0.15418 nm, U = 30 kV, I = 10 mA) and 5 rpm rotation. The step size was set to of 0.02025° (2θ) and the overall data were acquired in the angular range of 5°–80°. To evaluate the obtained data, X’Pert HighScore Plus software (2017, PANalytical, Almelo, The Netherlands) was used.

To study the surface morphology and microstructure of the prepared samples, scanning electron microscopy (SEM) was used (Tescan MAIA3, TESCAN Brno, s.r.o., Brno, Czech Republic).

The elemental composition and mapping were characterized using an energy-dispersive spectroscopy (EDS) analyzer (X-Max150, Oxford Instruments, High Wycombe, UK) with a 20-mm^2^ SDD (silicon drift detector) detector and AZtecEnergy software (Oxford instruments, High Wycombe, UK). The sample was set on a carbon conductive tape to ensure the conductivity of the experiments. For both SEM and SEM-EDS analysis, the electron beam was set to 10 kV.

To study the used carbon-based nanomaterials, HR-TEM was performed using an EFTEM Jeol 2200 FS microscope (Jeol, Tokyo, Japan). A 200 keV acceleration voltage was used for the measurement. The sample preparation was attained by drop-casting the suspension (1 mg∙mL^−1^ in isopropyl alcohol) on a TEM grid (Cu; 200 mesh; Formvar/carbon) and then drying in a vacuum dryer at 25 °C and p/p0 = 0.2.

The BET specific surface was measured using a sorption analyzer NOVAtouch LX2 (Quantachrome Instruments, Boynton Beach, Florida, USA). The sample was outgassed for 10 h at 100 °C under high vacuum. A nitrogen-cooled (77 K) detector was used for the evaluation of the results using BET (Brunauer, Emmett and Teller) and Kelvin equations. The Quantachrome software was used for evaluation of measured data and to recalculate the measured value to m^2^ per 1 g.

To verify the distribution and presence of applied oxidized nanoadditives and to reveal the main precipitated phases and bonds among the chemical forming the hardened structure of the researched composites, the MIR (mid-infrared) spectra were collected using ATR (attenuated total reflection) technique. The MIR spectra were obtained after 32 scans that were conducted using the FT-IR spectrometer Nicolet 6700 (Thermo Fisher Scientific, Waltham, MA, USA), which operates with spectral resolution 4 cm^−1^ at the spectral range 4000–400 cm^−1^. The analyzed samples were cut from the original prisms, dried in a vacuum drier and then mechanically crushed and homogenized with a ball grinder MM 400 (RETSCH, Haan, Germany). Finally, they were pulverized in an agate mortar.

The tests of the hardened composites were done for 28 days matured samples. The experimental campaign was proposed in such a way to evaluate the co-doping effect of the oxidized nanoadditives on the structural, mechanical, hygric, and thermal parameters on MOC-based matrix. For each composite mixture, at least 5 samples were subjected to the particular tests. To measure the dry bulk density, the casted prisms 40 mm × 40 mm × 160 mm were dried in a vacuum drier at 50 °C until their constant mass was achieved. The dry bulk density *ρ*_b_ (kg·m^−3^) was then measured as prescribed in the EN 1015-10 [60]. The fragments extracted from the inner part of the crushed prisms were analyzed in a helium pycnometer Pycnomatic ATC (Porotec, Hofheim, Germany) to determine their specific density *ρ*_s_ (kg·m^−3^). The total open porosity was calculated from the bulk density and specific density values [61]. The composites microstructure was studied on a mercury porosimetry principle using porosimeters of Pascal series, Pascal 140 and Pascal 440 (Thermo Fisher Scientific, Waltham, MA, USA). The pore size was evaluated from the mercury pressure using the Washburn–Laplace equation and the cylindrical capillaries were assumed. The investigated parameters were pore size distribution curves, total pore volume, average and modal pore diameter and pore surface area. The dry samples mass in microstructural analysis was about 2 g. The compressive strength *f*_c_ (MPa), flexural strength *f*_f_ (MPa), and the dynamic modulus of elasticity *E*_d_ (GPa) were the tested mechanical parameters. The mechanical strength tests were performed in compliance with the EN 1015-11 [62]. The flexural strength was measured on the casted prisms in a three-point bending strength test arrangement. On the rest of broken prisms, the compressive strength was assessed. The loaded cross section in the uniaxial strength tests was 40 × 40 mm^2^. The stiffness of MOC composites was characterized by the dynamic modulus of elasticity, which was tested by the Vikasonic apparatus (Schleinbinger Geräte, Buchbach, Germany) operating on a frequency of 54 kHz. The specimens were 40 × 40 × 160 mm prisms and were measured along their longitudinal axis. A capillary absorption test was used to evaluate the water transport and storage in the examined composites and to quantify the effect of nanoadditives usage on the deceleration and retardation of water ingress. The measured parameters were water absorption coefficient *A*_w_ (kg·m^−2^·s^−1/2^), 24-h water absorption *W* (kg·m^−2^), and 24-h water absorption *W*_a_ (wt. %). These were obtained in accordance with the EN 1015-18 [63]. The samples used in the water absorption experiment were prisms with a size of 40 × 40 × 160 mm. As the nano-doping of the MOC matrix affects its porous structure, and thus heat transport and storage, thermal conductivity *λ* (W·m^−1^·K^−1^), thermal diffusivity *a* (m^2^·s^−1^), and volumetric heat capacity *c*_v_ (J·m^−3^·K^−1^) of the matured samples were tested using a Hot Disk TPS 1500 (Hot Disk AB, Göteborg, Sweden) apparatus equipped with the Kapton-insulated sensor having a radius of 6.4 mm. The temperature of the thermal parameters test was (23 ± 2) °C. Before the measurement, the prismatic samples having a size of 40 × 40 × 50 mm were dried in a vacuum at 50 °C. The used experimental techniques and conducted test are together with the expanded combined uncertainties of the particular investigated parameters summarized in Table 3.

## 3. Results and Discussion

**Figure 3:**
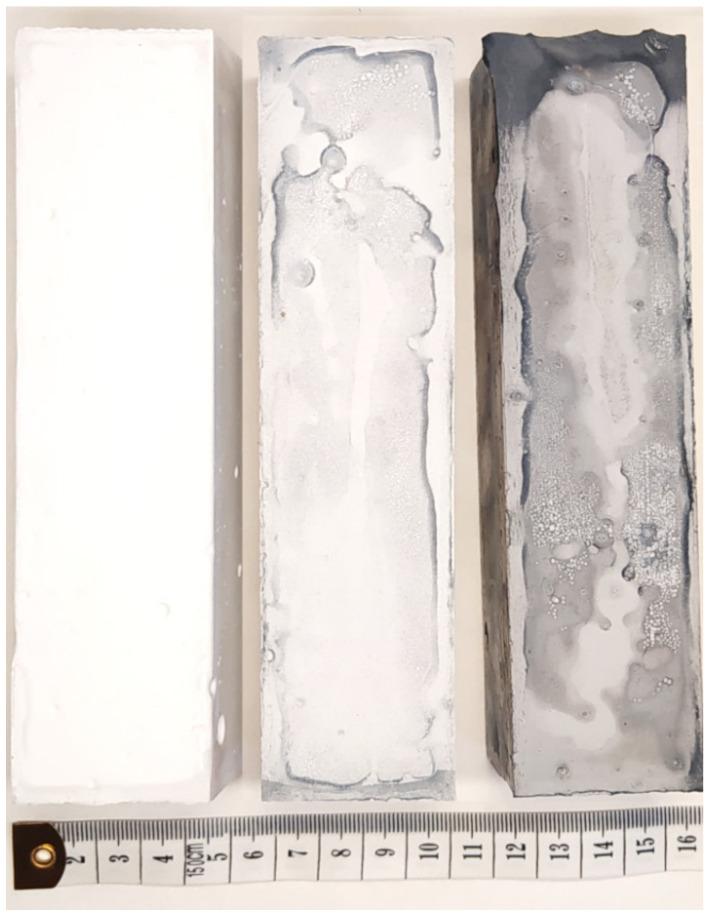
28-days specimens: MOC-REF, MOC-GO-OMWCNT-0.1, MOC-GO-OMWCNT-0.2 (from left).

We prepared three sets of samples and we compared their properties. Samples were marked as MOC-REF (reference with no added carbon nanostructures), MOC-GO-OMWCNT-0.1 (with 0.1 wt. % of GO and 0.1 wt. % of OMWCNT) and MOC-GO-OMWCNT-0.2 (with 0.2 wt. % of GO and 0.2 wt. % of OMWCNT). The hardened specimens are presented in Figure 3.

**Figure 4:**
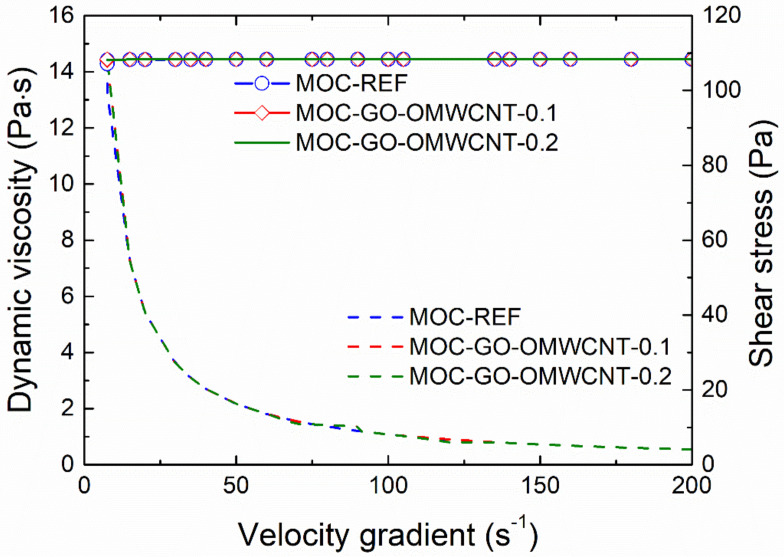
Dynamic viscosity and shear stress of fresh composite mixtures.

The effect of the application of nanoadditives on the rheology of fresh composites mixtures was studied using a rotation viscometer. The dynamic viscosity (dash lines) and the shear stress plotted in the dependence on the velocity gradient are graphed in Figure 4. As only minimum differences in investigated rheological parameters were recorded, the effect of the incorporation of GO and OMWCNTs can be considered as negligible, which enables one to design and develop multiscale composites with improved functional and technical properties. As used nanoadditives have no adverse effect on the workability of fresh composites, it is possible to incorporate different types of fillers, aggregates and reinforcing fibers in composite mixture that would bring other advanced parameters of the materials on nano-doped MOC basis.

**Figure 5:**
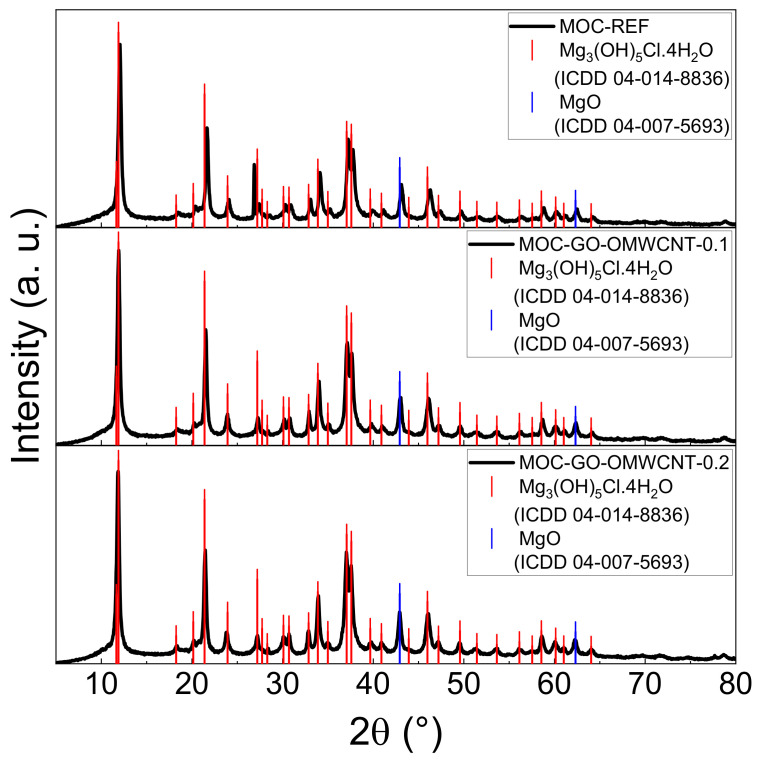
The XRD patterns of the samples MOC-REF (top), MOC-GO-OMWCNT-0.1 (middle) and MOC-GO-OMWCNT-0.2 (bottom).

To confirm the phase composition of the hardened composites, XRD analysis was used. The diffraction patterns of the three types of samples (see Figure 5) are all similar due to the presence of the two required crystalline phases—MOC Phase 5 (ICDD 04-014-8836) with the main reflection at 2θ = 11.9° and MgO (ICDD 04-007-5693) with its characteristic reflection at 2θ = 40.9°, which works as a filler in the samples. The carbon-based nanomaterials used as dopants are not visible in the diffraction patterns due to their low content.

**Figure 6:**
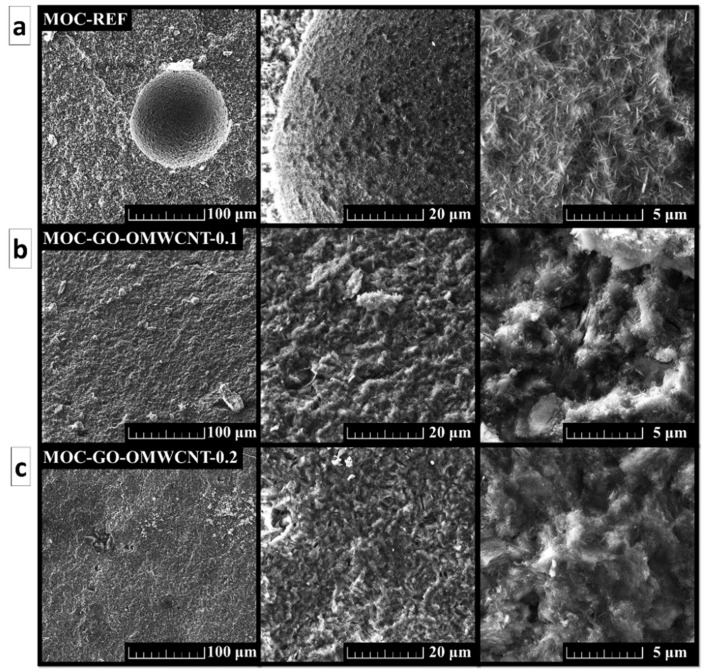
The SEM micrographs of (**a**) MOC-REF, (**b**) MOC-GO-OMWCNT-0.1 and (**c**) MOC-GO-OMWCNT-0.2.

The SEM was used in order to study the morphology of the prepared set of samples. The micrographs show in Figure 6 show a compact structure of the samples without cracks. For the sample MOC-REF, the typical needle-shaped crystals were visible, especially in the area of air-bubbles which formed in the suspension during mixing. The presence of needle-shaped crystals in the samples containing GO and OMWCNTs was also confirmed; however, these crystals had less distinct shape with blurred edges and, overall, were shorter and wider than the ones of the reference sample.

**Figure 7:**
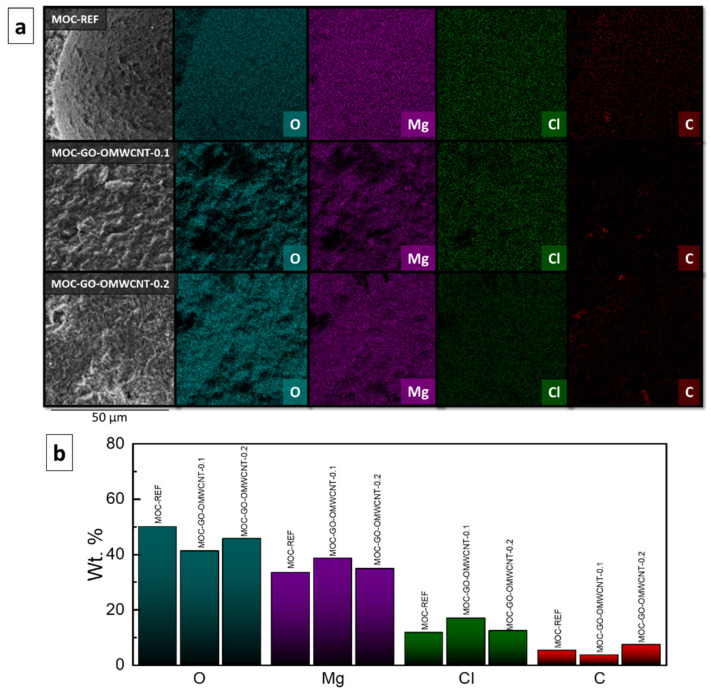
(**a**) The elemental maps and (**b**) the elemental composition (in wt. %) of the samples MOC-REF, MOC-GO-OMWCNT-0.1 and MOC-GO-OMWCNT-0.2.

The elemental maps (Figure 7a) and the elemental composition in wt. % (Figure 7b) of the prepared composites were provided by EDS. The elemental maps showed good dispersion of each element present in the samples. The quantitative part of the EDS analysis showed the content of each element for each sample. For the sample MOC-REF, the content of the elements was 50.0 wt. % of O, 33.3 wt. % of Mg, 11.6 wt. % of Cl and 5.1 wt. % of C. The carbon was present probably due to the reaction of MOC with the atmospheric CO_2_, during which magnesium carbonate phases such as chlorartinite were formed. The sample MOC-GO-OMWCNT-0.1 contained 41.2 wt. % of O, 38.5 wt. % of Mg, 16.8 wt. % of Cl and 3.5 wt. % of C. The third sample, MOC-GO-OMWCNT-0.2, contained 45.7 wt. % of O, 34.8 wt. % of Mg, 12.2 wt. % of Cl and 7.2 wt. % of C. The increase of the content of C is well visible between the samples containing 0.1 wt. % of each dopant and the samples containing 0.2 wt. % of GO and OMWCNTs. The increase also corresponded with the expected value.

**Figure 8:**
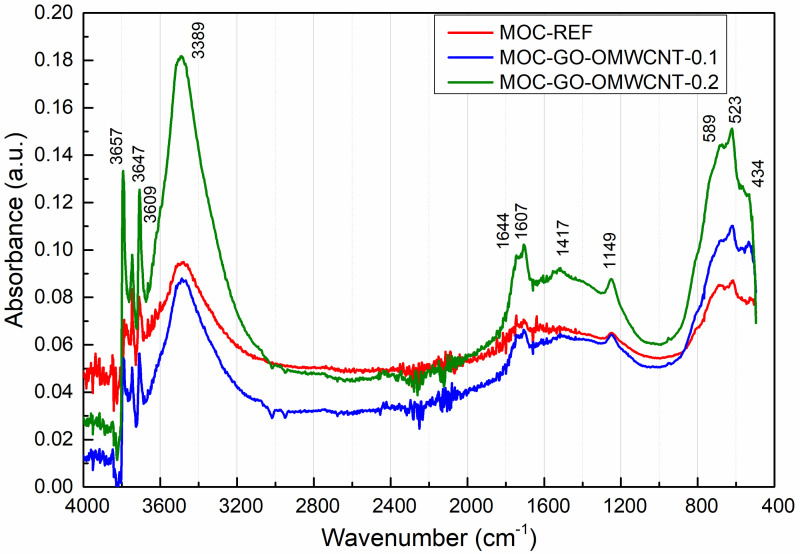
The collected MIR spectra of tested composites in the range of 400–4000 cm*^−^*^1^.

Figure 8 presents the collected MIR spectra of the analysed MOC composites. The assignment of the major absorption bands is summarized in Table 4. We can recognize characteristic bands corresponding to the basic vibrations of H_2_O and the lattice vibrations of MgCl_2_ and Mg(OH)_2_. The presence of the carbon nanotubes and graphene was significantly observed at 1607 cm*^−^*^1^, where the stretching vibration of C=C bond in the MIR spectrum could be identified [64,65]. The rising calculated peak area confirmed the presence of graphene oxide and carbon nanotubes. Namely, the peak area of the C=C bond stretching vibration was 0.093; 0.3168 and 0.6073 in the case of MOC-REF, MOC-GO-OMCWNT-0.1, and MOC-GO-OMCWNT-0.2, respectively. This data corresponds with the composition of composites and their mechanical parameters; it is also in an agreement with the relative brucite amount, which is manifested at 3695 cm^−1^ as the stretching vibration of the O-H group [66]. The content of brucite was mounted in the order MOC-GO-OMCWNT-0.2, MOC-GO-OMCWNT-0.1 and MOC-REF.

The spectral data presented as curves bear resemblance to each other; significant bands could be observed. In the range of 3300–3700 cm*^−^*^1^, the stretching vibrations O-H bond could be identified. At 1644 cm*^−^*^1^ and 1149 cm*^−^*^1^, the band could be assigned to O-H bending vibrations in MgCl_2_·8H_2_O. The broad band at 1417–1446 cm^−1^ was coming from the bending vibrations of O-H bonds in the hydroxyl group in Mg(OH)_2_ and the stretching vibration of C=O bond in magnesite and chlorartinite [67]. In the range below 600 cm^−1^, the series of the bands could be attributed to the lattice vibrations of Mg-O/Mg-Cl bonds and the stretching vibration of the Mg-O cubic structure.

The basic structural parameters of 28-days samples are summarized in Table 5. The mean values from the measurement of 5 samples of each examined composites are presented together with their expanded combined uncertainties. The co-doping of the control matrix by GO and OMWCNTs resulted in the increased bulk density and specific density; this densification led to the significant drop in porosity, which was approx. 15.8% for MOC-GO-OMWCNT-0.1 and 28.9% for MOC-GO-OMWCNT-0.2, respectively. Quantitatively, the researched composites exhibited really low porosity; thus, their high mechanical strength and durability may be tanticipated.

**Figure 9:**
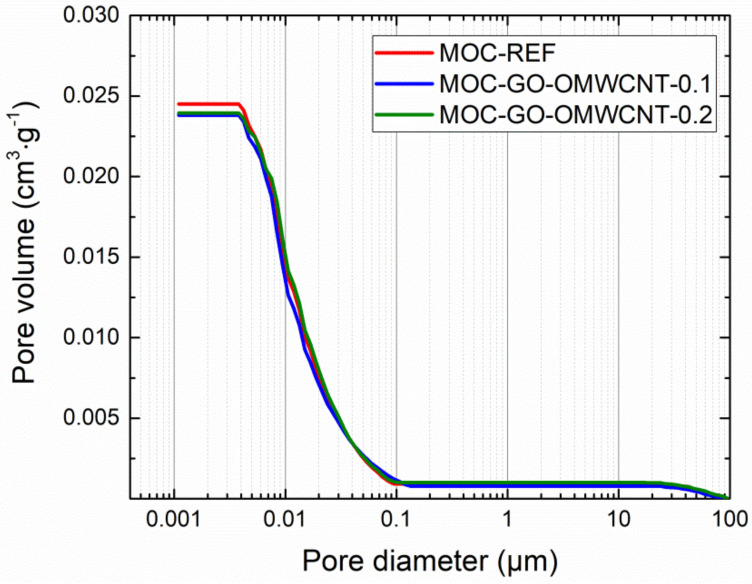
Pore size distribution—cumulative curves.

**Figure 10:**
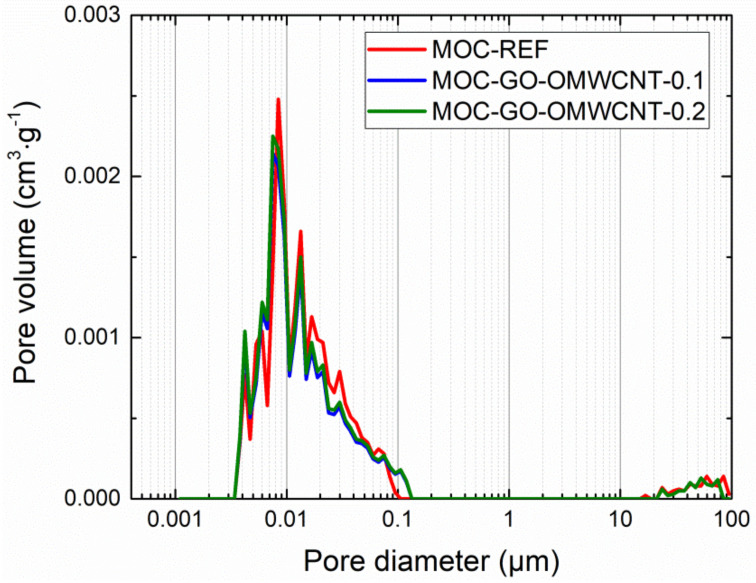
Pore size distribution—incremental curves.

In Table 6, the micro-structural parameters obtained by the mercury intrusion porosimetry (MIP) analysis are presented. The cumulative and incremental pore size distribution curves are displayed in Figure 9 and Figure 10. MIP microstructural data gives evidence of the densifying and solidification effect of co-doping of MOC matrix by C-based nanoadditives. For all examined materials, the shape of the cumulative pore size distribution curves was almost similar. In the case of the incremental pore size distribution, the differences in pore size were more distinctive, especially in the pore diameter range from 0.015 µm to 0.084 µm. These pores can be classified according to the IUPAC terminology [68] as macro-pores (>50 nm) and meso-pores (2–50 nm). Based on the pore size classification established in cement research, these pores are responsible for mechanical strength, permeability and, thus, frost resistance [69]. In this respect, one can simply estimate improvement in the mechanical performance and reduced water ingress by the co-doping of the MOC matrix.

**Figure 11:**
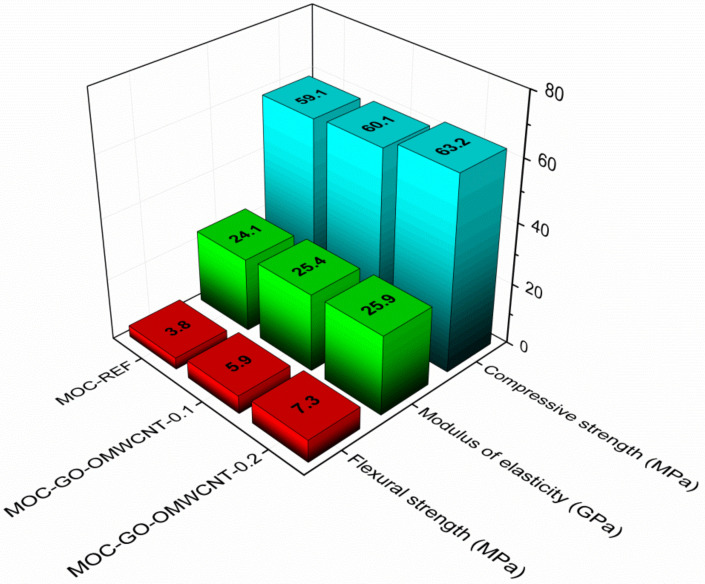
Mechanical properties of the 28-days matured samples.

The accessed mechanical parameters are presented in Figure 11. All materials exhibited high strength and stiffness that corresponded well with the nature of the MOC matrix [70,71] and its low porosity. It is evident that the pore structure had a great impact on material’s physical properties; thus, the lower the porosity and the smaller the pore number and the radii are the better the mechanical performance of the composite will be. As the compressive strength was affected by the nanoadditives to a low extent only, the combination of a high strength of graphene oxide [72,73], the bridging and interlocking effect of OMWCNTs [74], and the micro-pores filling resulted in a great improvement of flexural strength and toughening of the co-doped composites. The increase in the flexural strength was approx. 55.3% for MOC-GO-OMWCNTs-0.1 and 92.1% for MOC-GO-OMWCNT-0.2. In this respect, one must consider that only low dosage of nano-dopants was used, and further enhancement in the mechanical performance can be anticipated in the case of a higher content of nanoadditives incorporated in the composite mixture.

The water transport and storage properties obtained for the hardened composites are given in Table 7. In the comparison with the capillary porous materials [75,76,77], the water absorption coefficient and 24-h water absorption values were much lower; they were further reduced by the application of the nanoadditives. For example, the drop in *A_w_* was ~24% for MOC-GO-OMWCNT-0.1 and ~28% for MOC-GO-OMWCNT-0.2. Accordingly, 24-water absorption was reduced by 25% and 39%, respectively. Similarly, as in the case of mechanical parameters, the reduction in water ingress was assigned to the drop in the volume of pores accessible for water, total reduced pore number and an average pore size. The limited wettability of the nano-doped composites gives presumption of their durability in terms of moisture-induced damage and allows one to classify the applied carbon-based dopants as water repellant admixtures for the MOC matrix. In combination with inorganic fillers, e.g., on active SiO_2_ basis, advanced, multifunctional MOC composites can be further designed and developed, meeting the technical, functional and durability criteria of the construction materials intended for specific application purposes.

The thermal parameters measured on the transient hot disk technique principle are introduced in Table 8. Quantitatively, the values of the thermal conductivity were high, typical for dense inorganic materials, such as low porous concrete [78]. High thermal conductivity of MOC materials has already been reported in our previous studies, see, e.g., [79]. The assessed thermal properties represent results of two parallel effects: (i) dropped porosity and, thus, increased density that enhances itself for heat transport and storage, (ii) unique properties of carbon-based nanoadditives that, in respect to their morphology, size and defects [80], exhibit high thermal conductivity and heat capacity [81,82]; they are temperature dependent and show changes in dimensionality [83]. Although it is impossible to distinguish which effect was the dominant in enhancement of the analyzed thermal parameters, co-doping nanoadditives have served as controlling agents of heat transport and storage, respectively. This might find application in MOC composites with low heat conductive phase change materials that could be effectively heated by the bridging effect of OMWCNTs and dispersed GO particles.

## 4. Conclusions

In this research, the combined effect of oxidized 1D and 2D carbon nanoadditives on the MOC Phase 5 was studied. The following advanced findings of the analysis of the 28-days hardened samples were highlighted:

(i). the micro- and macro-structural parameters analyses showed the base of the enhancement caused by the carbon-based nanoadditives that significantly decreased the porosity, which was reduced by ~15.8% for MOC-GO-OMWCNT-0.1 and ~28.9% for MOC-GO-OMWCNT-0.2;
(ii). the great drop of porosity led to the increase in the mechanical parameters, where the flexural strength values increased by 55.3% and 92.1% for the samples containing 0.1 and 0.2 wt. % of each nanoadditive, respectively;
(iii). as the volume of pores decreased due to their filling and interlocking with the nanoadditives, the water transport and storage properties were significantly lowered; thus, the improved water resistance of the MOC matrix can be anticipated—this effect of the nanodopants is crucial for the MOC-based composites, which are usually considered as materials with very poor water resistance;
(iv). the materials thermal conductivity was quite high due to the decreased porosity, but also as a result of adapting the thermal conductivities of the used GO and OMWCNTs, which are generally very high.

Based on the summarized results it was concluded, the used nanoadditives have significant potential in the field of design and the development of advanced and multifunctional MOC-based composites. However, the homogenization and ideal dispersion of the nanodopants must be improved in order to get even greater enhancement of the composite properties.

## Figures and Tables

**Table 1:** Elemental composition of OMWCNT and GO observed using EDS (in wt. %).

EDS wt. %	C	O	S	Ni	Al
OMWCNT	96.2	2.6	-	0.8	0.4
GO	65.7	32.6	1.7	-	-

**Table 2:** Mixture proportion: dosage of the particular components.

Mixture/Composite	Mass (g)				
	MgO	MgCl_2_·6H_2_O	H_2_O	GO	OMWCNT
MOC-REF	553.6	399.0	247.3	-	-
MOC-GO-OMWCNT-0.1	552.5	398.2	246.9	1.2	1.2
MOC-GO-OMWCNT-0.2	551.4	397.4	246.4	2.4	2.4

**Table 3:** The expanded combined uncertainties (ECU) of the applied testing methods.

Material Parameter	Symbol	Unit	ECU (%)	Method/Standard
Bulk density	*ρ*_b_	(kg·m^−3^)	1.4	EN 1015-10
Specific density	*ρ*_s_	(kg·m^−3^)	1.2	Helium pycnometry
Total open porosity	*Ψ*	(-)	2.0	Gravimetry/pycnometry
Flexural strength	*f*_f_	(MPa)	1.4	EN 1015-11
Compressive strength	*f*_c_	(MPa)	1.4	EN 1015-11
Dynamic modulus of elasticity	*E*_d_	(GPa)	2.3	Ultrasonic pulse velocity
Water absorption coefficient	*A*_w_	(kg·m^−2^ s^−1/2^)	2.3	EN 1015-18
24-h water absorption	*W*	(kg·m^−2^)	1.2	EN 1015-18
24-h water absorption	*W*_a_	(wt. %)	1.2	EN 1015-18
Thermal conductivity	*λ*	(W·m^−1^·K^−1^)	2.3	Hot disk
Thermal diffusivity	*a*	(m^2^·s^−1^)	2.6	Hot disk
Volumetric heat capacity	*c*_v_	(J·m^−3^·K^−1^)	2.6	Hot disk

**Table 4:** Assignments of the major absorption bands identified in MOC composites.

Wavenumbers (cm^−1^)	Assignment
3695	stretching (ν) vibration of O-H in Mg(OH)_2_
3677	stretching (ν) vibration of O-H in crystalline hydroxyl
3647	stretching (ν) vibration of O-H in silicate hydrates
3611–3609, 3585	stretching (ν) vibration of H-O-H in MgCl_2_·8H_2_O
3388–3391	stretching (ν) vibration of H-O-H in H_2_O
1644, 1149	bending (δ) vibration of H-O-H in MgCl_2_·8H_2_O
1607	stretching (ν) vibration of C=C
1417, 1446	stretching (ν) C=O in MgCO_3_
1455–1423	stretching (ν) O-H in C-S-H and M-S-H phase,
592–584	deformation (δ) and stretching (ν) lattice vibrations of Mg-Cl/Mg-O
517–524, 457	translation vibrations of Mg/Mg-O, Mg-OH, and stretching (ν) vibration of O-Si-O in diatomite
429	bending (δ) vibration of O-Si-O in diatomite
414	vibrational modes of the lattice showing the Mg-O/Mg^2+^, O/O-Mg-O/O-Mg^2+^-O bonds

**Table 5:** Macro-structural parameters of 28 days matured composites.

Composite	Bulk Density *ρ_b_* (kg∙m^−3^)	Specific Density *ρ_s_* (kg∙m^−3^)	Total Open Porosity *Ψ* (%)
MOC-REF	1785 ± 25	1855 ± 22	3.8 ± 0.1
MOC-GO-OMWCNT-0.1	1801 ± 25	1861 ± 22	3.2 ± 0.1
MOC-GO-OMWCNT-0.2	1812 ± 25	1862 ± 23	2.7 ± 0.1

**Table 6:** Microstructural parameters of 28 days matured composites.

Composite	Average Pore Diameter (µm)	Median Pore Diameter (µm)	Total Pore Surface Area (m^2^·g^−1^)	Total Pore Volume (cm^3^·g^−1^)
MOC-REF	0.0113	0.0135	10.536	0.0298
MOC-GO-OMWCNT-0.1	0.0105	0.0127	9.258	0.0240
MOC-GO-OMWCNT-0.2	0.0103	0.0111	8.605	0.0238

**Table 7:** Hygric parameters of 28 days matured composites.

Composite	Water Absorption Coefficient *A*_w_ (kg∙m^−2^∙s^−1/2^)	24-h Water Absorption *W* (kg∙m^−2^)	24-h Water Absorption *W*_a_ (wt.%)
MOC-REF	2.5 × 10^−3^ ± 6 × 10^−5^	1.071 ± 0.013	0.9801 ± 0.1
MOC-GO-OMWCNT-0.1	1.9 × 10^−3^ ± 4 × 10^−5^	0.803 ± 0.009	0.6313 ± 0.1
MOC-GO-OMWCNT-0.2	1.8 × 10^−3^ ± 4 × 10^−5^	0.651 ± 0.008	0.5742 ± 0.1

**Table 8:** Heat transport and storage parameters of 28 days matured composites.

Composite	Thermal Conductivity *λ* (W∙m^−1^∙K^−1^)	Thermal Diffusivity *a* × 10^−6^ (m^2^·s^−1^)	Volumetric Heat Capacity *c*_V_ × 10^6^ (J·m^−3^·K^−1^)
MOC-REF	1.276 ± 0.029	0.493 ± 0.013	2.588 ± 0.067
MOC-GO-OMWCNT-0.1	1.322 ± 0.030	0.494 ± 0.013	2.675 ± 0.070
MOC-GO-OMWCNT-0.2	1.334 ± 0.031	0.474 ± 0.012	2.815 ± 0.073

## Data Availability

The data presented in this study are available on request from the corresponding author. The data are not publicly available due to privacy.
